# Cancer research activity in the Arab world: a 15-year bibliometric analysis

**DOI:** 10.1186/s42506-022-00120-6

**Published:** 2022-11-17

**Authors:** Marc Machaalani, Jad El Masri, Lemir Majed El Ayoubi, Bassam Matar

**Affiliations:** 1 Faculty of Medical Sciences, Lebanese University, Beirut, Lebanon; 2 Faculty of Medical Sciences, Neuroscience Research Center, Lebanese University, Beirut, Lebanon; 3 Faculty of Medical Sciences, Department of Internal Medicine, Division of Haematology and Oncology, Lebanese University, Beirut, Lebanon

**Keywords:** Bibliometric analysis, Arab countries, Cancer, Research productivity

## Abstract

**Background:**

The Arab region comprises 22 countries located in the Middle East and North Africa, sharing cultural and linguistic ties. Arab countries have continued to lag in terms of biomedical research compared to other nations for several past decades. Cancer is a major public health concern, being the second leading cause of death globally. Given that high research activity on cancer reflects positively on screening programs, awareness, and clinical practice, this article aimed to examine the activity and trend of cancer research in the Arab world between 2005 and 2019.

**Methods:**

Between 2005 and 2019, the number of cancer-related articles published by each Arab country, and regarding 27 different types, was assessed using the PubMed database. Numbers were normalized with respect to each country’s average population and average Gross Domestic Product (GDP).

**Results:**

Arab countries contributed to 1.52% of total cancer publications. The number of cancer publications has steadily grown since 2005, with the last 7 years alone witnessing 75.69% of the total Arab cancer-related publications. In terms of publications per million persons, Qatar ranked first (393.74 per million persons), while in terms of publications per national GDP, Egypt ranked first (464.27 per billion US dollars). Breast, liver, and colorectal cancers had the highest numbers of all Arab cancer-related publications, while testicular, vulvar, and gallbladder cancers had the least.

**Conclusions:**

This paper pools information and insight for scientists, clinicians, funders, and decision-makers on the actualities and developments of cancer research in the Arab world. Addressing the barriers facing cancer research remains a cornerstone in the plan to improve the Arab world’s output and contribution to the field of oncology.

## Background

Descriptions of cancer date back to 3000 B.C. and the earliest evidence of the disease was found among mummies in ancient Egypt and fossilized bone tumors [1]. Nowadays, cancer remains a major public health concern. In 2020, it is estimated that the global cancer burden rose to 19.3 million new cases and 10.0 million deaths. Approximately one in 5 people worldwide develop cancer during their lifetime, whereas one in 8 men and one in 11 women die from the disease [2]. This makes cancer the second leading cause of death globally, behind heart disease, according to the World Health Organization (WHO) [3].

The Arab region comprises 22 countries located in the Middle East and North Africa. With over 400 million inhabitants in total, these countries made up 5.5% of the world population and contributed 3.2% to the world gross domestic product (GDP) in 2019. While they share cultural and linguistic ties, they are markedly diverse in terms of education, economic development, and healthcare infrastructure [4]. Particularly, discrepancies within the Arab world are significant when it comes to scientific research output [5–10]. Furthermore, Arab countries have continued to lag behind in terms of biomedical research compared to other nations for several past decades [5, 11].

Cancer incidence varies remarkably within and between Arab populations [12]. Given that high research activity on cancer reflects positively on screening programs, awareness, epidemiological data, and clinical practice, interest and investment in research have been steadily growing. However, cancer research output among Arab countries is yet to be comprehensively assessed. Within this context, this article aimed to examine the activity and trend of cancer research in the Arab world between 2005 and 2019.

## Methods

### Database and search strategy

Screening for cancer-related publications was done for all 22 Arab countries, for 15 years, between 2005 and 2019. These countries include Algeria, Bahrain, Comoros, Djibouti, Egypt, Iraq, Jordan, Kuwait, Lebanon, Libya, Mauritania, Morocco, Oman, Palestine, Qatar, Saudi Arabia, Somalia, Sudan, Syria, Tunisia, United Arab Emirates, and Yemen.

Using Boolean operator, we searched PubMed for:

- MeSH Term: “cancer” OR “neoplasm” OR “tumor” OR “carcinoma” OR “adenocarcinoma” OR “leukemia” OR “leukaemia” OR “sarcoma” OR “lymphoma” OR “malignant” OR “oncology” OR “metastasis” OR “oncogene” OR “chemotherapy”.
- Affiliation: Arab countries, representing authors’ countries. We excluded cities in the USA called Lebanon using Boolean operator NOT. Regarding Palestine, we used West Bank and Gaza.
- MeSH Date: 2005–2019.

Inclusion and exclusion criteria are as follows:

- All types of articles were included.
- Articles with no authors from the targeted countries were excluded.
- Articles published before 2005 or after 2019 were also excluded.

**Fig. 1:**
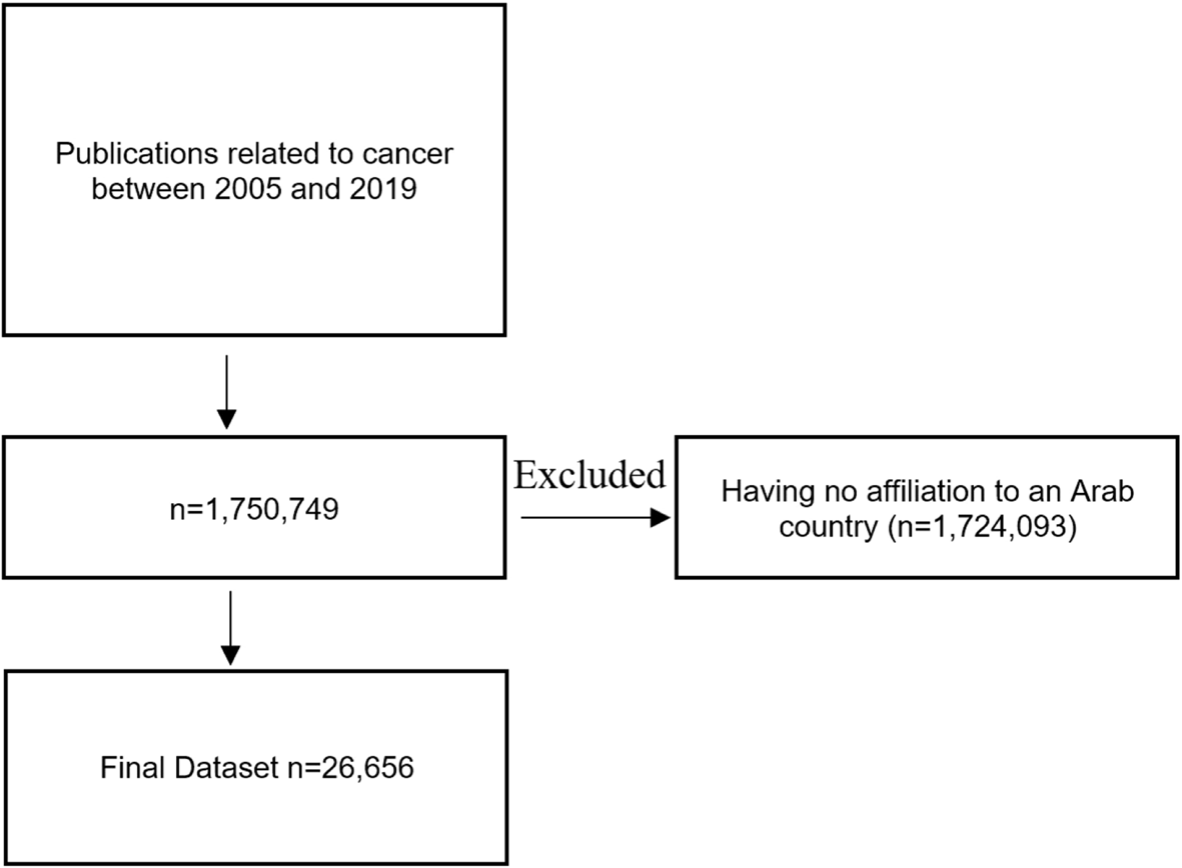
Identification and selection of publications related to cancer

Figure 1 is a PRISMA chart that clarifies the selection process of publications that were included in this study.

### Interpretation and comparison

The average population was calculated for each country between 2005 and 2019 using the 2019 World Prospect Population (WPP-2019). Besides, the average GDP was calculated in the same period from the World Bank [4, 13]. For each country, the number of publications per 1,000,000 persons was calculated, as well as per GDP. Similar approaches were used in other bibliometric analyses [5–7]. The number of publications for 27 types of cancer was also quantified, using similar methods to those of a study assessing the representation of cancer in the medical literature [14].

### Statistical analysis

Data were analyzed using SPSS (Statistical Package for Social Sciences) version 22, to assess the strength of the relation between the number of cancer publications and both the average population and average GDP.

## Results

Our findings revealed that a total of 26,656 cancer-related studies were published in the Arab world between 2005 and 2019, representing 13.4% of the total Arab biomedical research papers, and 1.52% of the world’s cancer-related studies published during that period. Overall, the total Arab publications accounted for 1.42% of the world’s biomedical literature during these 15 years (Table 1).

**Table 1:** Number and percentage of cancer-related publications in Arab countries

Country	Number of publications on cancer	Number of total publications	% cancer of total
Egypt	8917	53,290	16.73
Saudi Arabia	6589	53,898	12.23
Lebanon	2019	12,227	16.51
Tunisia	1811	14,633	12.38
Jordan	1327	10,817	12.27
Morocco	1276	7618	16.75
United Arab Emirates	932	9731	9.58
Qatar	880	8265	10.65
Kuwait	708	5646	12.54
Oman	484	4835	10.01
Iraq	476	4407	10.80
Algeria	314	4062	7.73
Sudan	271	3069	8.83
Syria	175	1356	12.91
Bahrain	170	1460	11.64
Libya	131	997	13.14
Yemen	129	1178	10.95
Palestine	39	1042	3.74
Mauritania	3	108	2.78
Somalia	3	94	3.19
Djibouti	2	97	2.06
Comoros	0	39	0.00
**Total**	26,656	198,869	13.40
**Worldwide**	1,750,749	13,995,404	12.509

Among the 22 Arab countries, only Comoros had no cancer-related publication during these 15 years. Moreover, Djibouti, Mauritania, and Somalia each had fewer than 4 publications, accounting for no more than 0.012% of total Arab cancer-related studies. On the other hand, Egypt published the biggest number of cancer-related studies (8917) among Arab countries, followed by Saudi Arabia (6589). These two countries alone collectively contributed to ~58.2% of the total cancer-related publications among Arab countries during the studied period.

As for each country’s share of cancer-related papers out of its total research, Morocco was in the first position, with 16.75% of its total publications being cancer-related, just ahead of Egypt (16.73%) and Lebanon (16.51%).

**Fig. 2:**
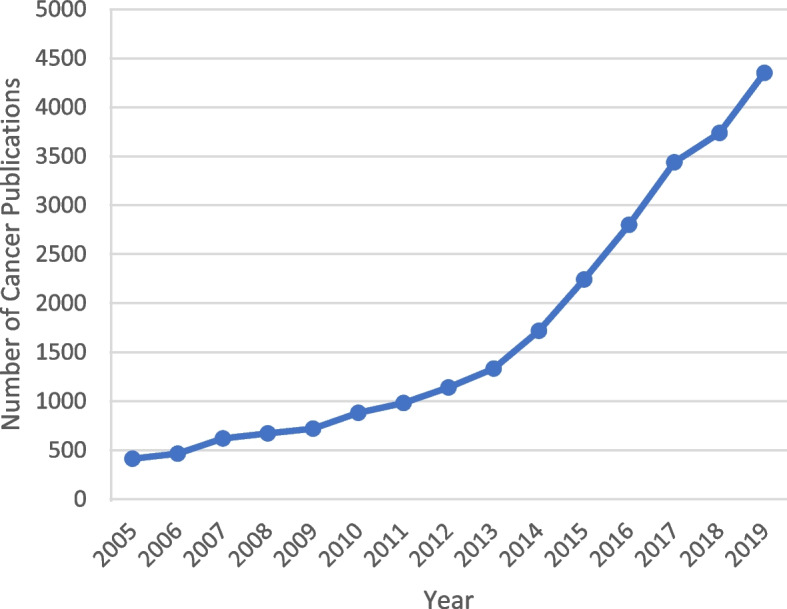
Line graph showing the number of total cancer-related publications in the Arab world each year

The number of cancer publications has steadily grown since 2005, with the last 7 years alone witnessing 75.69% of the total Arab cancer-related publications (Fig. 2).

In terms of publications per million persons, Qatar ranked first with a ratio of 394.74 publications per million persons, followed by Lebanon and Saudi Arabia (Table 2).

**Table 2:** Number of cancer-related articles per million persons

Country	Average population	Number of publications per million persons
Qatar	2,235,000	393.74
Lebanon	6,152,000	328.19
Saudi Arabia	31,459,000	209.45
Kuwait	3,588,000	197.32
Tunisia	11,653,000	155.41
Jordan	8,719,000	152.20
Oman	3,952,000	122.47
Bahrain	1,390,000	122.30
United Arab Emirates	8,901,000	104.71
Egypt	93,895,000	94.97
Morocco	35,797,000	35.65
Libya	6,739,000	19.44
Iraq	35,151,000	13.54
Syria	20,294,000	8.62
Palestine	4,592,000	8.49
Algeria	40,594,000	7.74
Sudan	39,399,000	6.88
Yemen	26,525,000	4.86
Mauritania	1,272,000	2.36
Somalia	1,335,000	2.25
Djibouti	939,000	2.13
Comoros	785,000	0.00

A significant relationship between the number of cancer-related publications and the average population was revealed (*P*<0.001), with *R*=0.7 suggesting a strong correlation. The number of cancer-related publications may therefore be considered to be increased by the average population (*R* squared was 0.49).

In terms of publications per national GDP, Egypt ranked first with a ratio of 464.27 publications per billion US$, far ahead of Lebanon in second place with 47.38 publications per billion US$ (Table 3).

**Table 3:** Number of cancer-related articles per GDP

Country	Average GDP (in billion US$)	Number of publications per billion US$
Egypt	19.21	464.27
Lebanon	42.62	47.38
Jordan	30.30	43.79
Tunisia	41.90	43.22
Syria	6.84	25.58
Morocco	95.27	13.39
Saudi Arabia	607.29	10.85
Oman	63.82	7.58
Qatar	134.00	6.57
Bahrain	28.76	5.91
Kuwait	132.53	5.34
Yemen	29.89	4.32
Sudan	68.65	3.95
Palestine	9.98	3.91
United Arab Emirates	323.86	2.88
Iraq	165.99	2.87
Libya	54.45	2.41
Algeria	169.76	1.85
Djibouti	1.71	1.17
Somalia	4.22	0.71
Mauritania	4.87	0.62
Comoros	0.97	0.00

A moderate (*R*=0.42) yet significant (*P*<0.001) correlation seemed to exist between the number of cancer-related publications and the average GDP. The number of cancer-related publications may be considered to be increased by the GDP (*R* squared was 0.17).

The publications on 27 types of cancers were tracked, and breast cancer was found to have the highest number of Arab papers among all cancer-related publications (2241). Colorectal and hepatic cancers each also had more than 1000 publications during the studied period, whereas testicular, vulvar, and gallbladder cancers had the least publications (< 60) (Table 4).

**Table 4:** Number of total publications per cancer type in the Arab world

Cancer	Number of publications
Breast	2241
Colorectal	1169
Liver	1017
Leukemia	972
Lung	761
CNS	753
Lymphoma	731
Uterine	562
Prostate	501
Mouth	468
Urinary, bladder	435
Ovarian	379
Thyroid	320
Kidney	296
Melanoma	292
Pancreases	253
Stomach	230
Hodgkin	157
Myeloma	140
Soft Tissue	136
Small Intestine	90
Esophageal	84
Laryngeal	81
Mesothelioma	68
Testicular	59
Vulvar	37
Gallbladder	23

Case reports were the most conducted types of studies in the Arab world (4060 publications). Different types of reviews made up of 4087 publications (3226 reviews, 452 systematic reviews, and 409 meta-analyses) (Table 5).

**Table 5:** Number of cancer-related publications in Arab countries according to the type of paper

Type of paper	Number of publications
Case report	4060
Reviews	3236
Clinical trials	2171
Letter	564
Systematic reviews	452
Meta-analysis	409
Commentaries	302
Observational	282
Editorials	157
Technical notes	0

## Discussion

Notwithstanding the heavy healthcare burden of cancer, research efforts in the field of oncology remain limited in the Arab world. Collectively, all 22 Arab countries contributed to a mere 1.52% of the world’s literature on cancer during the studied period. The overall trend revealed steady growth in the number of papers from the Arab world over the last 15 years. Egypt ranked first in terms of publications per average GDP, whereas Qatar ranked first concerning publications per average population. Comoros was revealed to have no cancer-related publications. The most reliable metric to assess and compare the research activity on cancer in Arab world countries was found to be the average population size which exhibited a strong correlation with the number of publications, whereas the correlation was weaker with GDP. This finding was echoed by another study on psoriasis research in the Arab world [15]; nevertheless, GDP was found to be the most accurate measure to assess stroke publications [16].

Several socioeconomic factors may play a role in hindering cancer publications and research activity in the Arab world. First of all, Arab funding of research is still relatively modest. In 2013, the gross expenditure on research and development (GERD) by the entire Arab world constituted only 1.0% of total global expenditures on research [17]. Indeed, Arab states with the least publications in cancer research were mostly low-income countries such as Comoros, Djibouti, Mauritania, and Somalia. These countries suffer from poor health services, widespread poverty, and a lack of education [18]. On the other hand, Gulf countries such as the United Arab Emirates (UAE), Saudi Arabia, and Qatar have been increasingly pouring oil revenues into technology and science research [19–21], which was markedly illustrated on February 9^th^, 2021, when the United Arab Emirates became the first Arab country and the fifth country worldwide to send a probe to Mars [22]. Deficient funding can impede the establishment of robust research infrastructures within Arab medical schools and medical centers. As a matter of fact, most Arab medical schools and hospitals remain patient-centered and clinically oriented, as only recently has a research culture been prioritized [20, 23].

Furthermore, wars, internal turmoil, and ongoing conflicts may further jeopardize research activity in the Arab world by channeling funds usually allocated for biomedical research activity towards military action and into fulfilling basic needs. War-torn countries also suffer from brain drain and insecure environments that can hamper research activity [24, 25]. This would thereby explain why Iraq, Libya, Palestine, Somalia, Sudan, Syria, and Yemen have fared relatively poorly in terms of research outcomes in the studied period.

In this study, Egypt had the highest productivity in publishing cancer-related research among Arab states. Major reasons include that the country boasts the biggest population among the studied countries [4], and one which suffers from alarmingly increasing rates of cancer [12, 26]. The most common diagnoses among the Egyptian population include liver cancer in males (33.6%) and breast cancer in females (32.0%) [27].

Concerning all the Arab world, breast, colorectal, and liver cancers garnered the most cancer-related publications. This finding is hardly surprising. Breast cancer is the most prevalent cancer in Arab countries, and its incidence keeps on increasing [28, 29]; liver cancer is a critical problem in males in Egypt and Saudi Arabia, the two leading countries in total cancer-related publications [12]; and some of the highest rates of colorectal cancer can be found among Gulf countries [30].

Cancer is one of the leading causes of death in the Arab world, and its incidence remains on the rise in this region [31, 32]. To the best of the authors’ knowledge, this is the first study assessing the Arab world’s contribution to the field of oncology and the distribution of publications regarding each type of cancer.

### Limitations

Despite its strengths, this bibliometric analysis should also be viewed in light of some limitations. The publications were obtained only from one database, PubMed, since it is the world’s largest medical library and contains only biomedical work. In contrast, when collecting studies from multiple databases, some publications may be counted more than once resulting in duplicate studies. Besides, only publications written in English were gathered in this study, therefore omitting papers in Arabic, French, or other languages. On this basis, the credibility of our results may be affected as the number of publications might be underestimated.

## Conclusions

Cancer research is a growing field in the Arab world and for valid reasons. Yet, the Arab contribution to the field of oncology remains a humble share of the world’s output due to diverse socioeconomic factors that impede research activity. Our paper pools background information for scientists, clinicians, funders, and decision-makers by providing insight on the actualities and trends of cancer research in the Arab world, and its distribution upon cancer types, thereby laying the framework for future developments.

## Data Availability

The datasets used and/or analyzed during the current study are available from the corresponding author on reasonable request.
